# From Symptomatic Therapies to Disease-Modifying Approaches for Neuronal Sodium Channel Disorders

**DOI:** 10.3390/ijms27010032

**Published:** 2025-12-19

**Authors:** Giorgia Dinoi, Ileana Canfora, Daniela D’Agnano, Brigida Boccanegra, Elena Conte, Annamaria De Luca, Antonella Liantonio, Vittorio Sciruicchio, Paola Imbrici

**Affiliations:** 1 Department of Pharmacy-Drug Sciences, University of Bari “Aldo Moro”, Via Orabona 4, 70125 Bari, Italy; giorgia.dinoi@uniba.it (G.D.); ileana.canfora@uniba.it (I.C.); brigida.boccanegra@uniba.it (B.B.); elena.conte@uniba.it (E.C.); annamaria.deluca@uniba.it (A.D.L.); antonella.liantonio@uniba.it (A.L.); 2 Children Epilepsy and EEG Center, San Paolo Hospital, ASL Bari, 70132 Bari, Italy; daniela.dagnano@gmail.com (D.D.); vittorio.sciruicchio@asl.bari.it (V.S.)

**Keywords:** sodium channels, Dravet syndrome, zorevunersen, ETX-101, elsunersen, fenfluramine

## Abstract

Variants in neuronal sodium channel genes are responsible for a spectrum of neurological disorders, including developmental and epileptic encephalopathies (DEEs), with considerable genetic and phenotypic heterogeneity and drug resistance. Gene variants can produce loss-, gain-, or mixed-function effects, resulting in complex genotype-phenotype correlations. Current treatments rely mainly on symptomatic polytherapy with antiseizure medications, with sodium channel blockers contraindicated in loss-of-function cases but beneficial in gain-of-function forms. Existing therapies often provide limited benefit or even no seizure control at all and fail to address developmental impairments, highlighting the need for novel approaches. Emerging strategies include antisense oligonucleotides, gene therapy, and selective small-molecule modulators, which have shown antiseizure potential in preclinical models and in initial clinical studies by modulating *SCN* gene expression and function. Additionally, pharmacological agents such as fenfluramine, stiripentol, and cannabidiol, although not acting directly on sodium channels, represent recognized therapeutic options for *SCN1A*-related Dravet syndrome. This review summarizes recent advances in approved and investigational treatments for sodium channel-related neurological disorders, highlighting the transition from symptomatic to precision therapies.

## 1. Introduction

**Figure 1:**
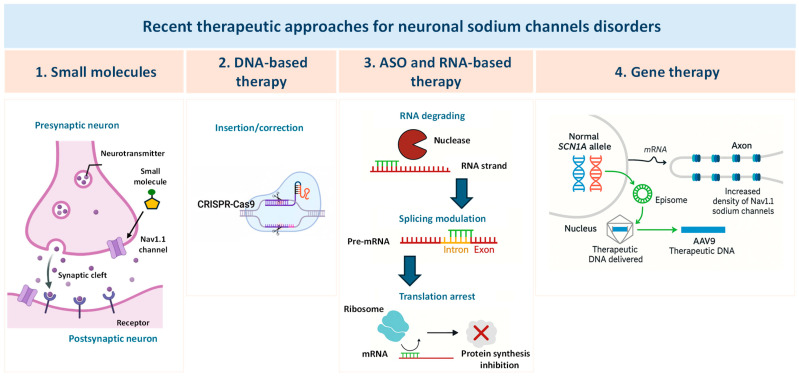
Emerging therapeutic strategies targeting neuronal sodium channel disorders. The image illustrates four main approaches: (1) Small molecules modulating ion channel activity at the synapse; (2) DNA-based therapy using CRISPR-Cas9 for gene correction; (3) Antisense oligonucleotides (ASOs) and RNA-based therapy including RNA degradation, splicing modulation, and translation arrest; (4) Gene therapy delivering therapeutic DNA via AAV9, leading to episomal expression and increased Nav1.1 channel density. See also Table 1.

Neuronal sodium channel genes, *SCN1A*, *SCN2A* and *SCN8A*, have been extensively involved in the pathophysiology of a broad spectrum of neurological disorders, including developmental and epileptic encephalopathy (DEE) in which epilepsy and neurodevelopmental comorbidities coexist [1]. Variants in these genes can result in loss-of-function (LOF), gain-of-function (GOF), or mixed LOF/GOF effects, leading to diverse and complex clinical phenotypes. In the absence of approved therapies that specifically target the underlying molecular defects, the current standard of care of sodium channel-related neuronal disorders mainly relies on polytherapy with antiseizure medications (ASMs), often in combination with drugs aimed at managing associated comorbidities. Importantly, sodium channel blockers (SCBs) are contraindicated in individuals carrying LOF variants but can be effective in those carrying GOF variants, and a specific treatment algorithm has been established for Dravet syndrome (DS), the most severe DEE linked to *SCN1A* mutations [2]. However, existing treatments have limited efficacy, often carry substantial side effects, and do not always address the neurodevelopmental impairments that extend beyond seizure control especially in *SCN*-related DEEs. This highlights an urgent need for novel therapeutic strategies [3]. Actually, the therapeutic management of DEEs is facing a paradigm shift, moving from a symptomatic treatment approach with classical ASMs to disease-modifying therapies aimed at producing a global improvement [4]. Emerging approaches for treating *SCN*-related neurologic disorders include antisense oligonucleotides (ASOs), gene therapy, and novel selective small molecules [5]. ASOs consist of a single-stranded base sequence that can act on specific RNA or DNA molecules, thereby modulating the expression of proteins encoded by the specific transcript. By addressing the underlying genetic cause of the disease, ASOs have the potential to achieve seizure freedom and potentially improve developmental comorbidities. Preclinical evidence suggests that selective modulation of *SCN1A*, *SCN2A* and *SCN8A* expression and function through ASOs targeting human mRNA or gene therapy could offer a promising solution for LOF and GOF variants [5,6]. Among other strategies, drug repurposing led to the repositioning of fenfluramine that emerged as a highly effective and well-tolerated treatment for seizures associated with DS, with a dual serotonergic and sigma-1 receptor activity. Clinical trials support fenfluramine’s role not only in seizure control but also in ameliorating the neurodevelopmental trajectory and in the reduction in SUDEP risk in DS [7,8]. Additional serotonergic modulators, such as bexicaserin and clemizole, are under investigation and represent potential therapeutic opportunities for patients with DEE. The exploration of novel drug targets and the growing understanding of the interplay between epilepsy and metabolic pathways have opened new avenues for therapies targeting metabolic enzymes, such as stiripentol and soticlestat, specifically for DS [7]. This narrative review summarizes some of the most recent therapeutic approaches approved or under investigation for the treatment of *SCN1A*-, *SCN2A*- and *SCN8A*-related neurologic disorders (Figure 1; Table 1).

## 2. *SCN1A*-DEE

The *SCN1A* gene encodes the α-subunit of the voltage-gated sodium channel Nav1.1, which plays a crucial role in the initiation and propagation of action potentials, particularly within fast-spiking GABAergic interneurons of the neocortex, hippocampus, and cerebellum. Mutations in *SCN1A* can result in LOF, GOF, or mixed functional effects and have been linked to a broad spectrum of neurological disorders with highly variable clinical features and treatment responses [1,28]. LOF variants have been classically associated with DS, a severe, drug-resistant DEE, as well as with milder forms such as genetic epilepsy with febrile seizures plus (GEFS+), which are more responsive to antiseizure medications. GOF variants, originally identified in familial hemiplegic migraine type 3 (FHM3), have more recently been linked to neonatal-onset epileptic encephalopathies with or without movement disorders, which often show a positive response to SCBs [29,30,31]. Mixed GOF/LOF variants have been described in early infantile DEEs characterized by neonatal seizures, profound developmental delay, and hyperkinesia [32]. Studies in mouse models have demonstrated that LOF in *Scn1a* leads to reduced inhibitory output from GABAergic interneurons, resulting in cortical hyperexcitability and seizures [33,34,35]. This impaired GABAergic signaling, including a delayed GABA switch, is also believed to contribute to the cognitive and behavioral comorbidities observed in patients [36,37]. As in many genetic disorders, the genotype-phenotype correlation in *SCN1A*-related epilepsy is often not straightforward. Clinical variability among individuals carrying the same variant, even within families, suggests a more complex disease mechanism involving genetic background, gene regulation, and neuronal network remodeling, beyond the primary ion channel dysfunction [38,39,40,41]. Therapeutic response in *SCN1A*-related epilepsies strongly depends on the functional nature of the causative variant. While, as said, SCBs (e.g., carbamazepine, phenytoin) may be effective in some GOF cases, they are contraindicated in LOF disorders such as DS, where they can exacerbate seizure activity [2]. Accordingly, the functional characterization of *SCN1A* variants is critical not only for variant classification but also for guiding treatment decisions. Based on preclinical research in animal models, the most beneficial therapeutic approaches in DS are based on drugs enhancing GABAergic transmission; commonly used medications include valproate, clobazam and stiripentol. More recently, cannabidiol and fenfluramine have been approved as adjunctive therapies in children of >2 years of age [2]. As mentioned before, given the limitations of current treatments, novel therapeutic strategies are under investigation. These include ASOs and gene therapy designed to upregulate functional *SCN1A* expression. Additionally, the interplay between epilepsy and metabolism has stimulated interest in therapies targeting also metabolic pathways, such as stiripentol and soticlestat, which offer alternative mechanisms to modulate neuronal excitability.

### 2.1. Stiripentol

Stiripentol is a structurally unique ASM that enhances central GABAergic transmission through multiple mechanisms. It prolongs the opening duration of GABA_A_ receptors and inhibits both GABA reuptake and metabolism, thereby increasing synaptic GABA availability and neuronal inhibition [42]. In the early 2000s, stiripentol received orphan drug designation and regulatory approval in Europe for the treatment of DS [43]. Currently, stiripentol is indicated specifically as an adjunctive therapy in combination with valproate and clobazam for patients with DS who are not adequately controlled with the two drugs alone. This combined therapy has demonstrated robust efficacy in reducing seizure frequency, including clinically meaningful (≥50%) and profound (≥75%) reductions in monthly convulsive seizure frequency (MCSF), in multiple clinical trials [43,44].

Beyond its well-known GABAergic mechanisms, preclinical studies have shown that stiripentol at a concentration of 500 μM inhibits lactate dehydrogenase (LDH), a key enzyme in the astrocyte-neuron lactate shuttle, responsible for the bidirectional conversion between lactate and pyruvate. Inhibition of LDH disrupts this metabolic coupling, suppressing neuronal hyperexcitability and contributing to reduce seizure susceptibility in a mouse model [9]. This emerging mechanism suggests a potential role for stiripentol in targeting metabolic pathways contributing to epileptogenesis.

The efficacy of stiripentol has also been explored in other DEEs beyond DS, including Lennox–Gastaut syndrome (LGS) and CDKL5 deficiency disorder. Across these studies, stiripentol was generally well tolerated, with the most common adverse effects being somnolence, anorexia, and weight loss [45]. Importantly, these adverse events were often reversible upon dose adjustment or discontinuation [46,47,48].

A recent comparative study has evaluated the efficacy and safety of stiripentol along other FDA- and EMA-approved therapies for DS, notably fenfluramine and cannabidiol. Stiripentol and fenfluramine demonstrated comparable efficacy in achieving ≥50% and ≥75% reductions in monthly convulsive seizure frequency (MCSF), with both agents significantly surpassing cannabidiol at all doses [49]. Importantly, stiripentol was the only agent showing a statistically significant advantage over both fenfluramine and cannabidiol in achieving complete seizure freedom from baseline. Furthermore, while the incidence of serious adverse events was similar within all three therapies, stiripentol exhibited a lower risk of treatment discontinuation due to adverse effects, particularly at its standard dose of 20 mg/kg/day [10,49].

These findings support current international consensus guidelines, which prioritize stiripentol and fenfluramine over cannabidiol in the adjunctive management of DS [2]. Therefore, stiripentol remains a mainstay therapy in DS, with growing interest in its broader applications across the epilepsy spectrum.

### 2.2. Fenfluramine

In 2020, fenfluramine received FDA and EMA approval as an adjunctive therapy to standard of care for seizures associated with DS and, later, LGS, for individuals aged 2 years and older [50]. Along with its active metabolite norfenfluramine, this drug acts primarily on the serotonergic system promoting serotonin (5-HT) release and selectively activating 5-HT_1D_ and 5-HT_2C_ receptors. In addition, a modulatory action on the sigma-1 receptor, a chaperone protein known to interact also with ion channels, has been supposed to contribute to its antiseizure effects. Thus, through this dual mechanism of action, fenfluramine potentiates the GABAergic transmission while reducing the glutamatergic one [51]. This drug was originally developed and approved as an anorectic agent due to its appetite-suppressing effects. However, by the late 1990s, safety concerns about cardiac valvulopathy and pulmonary arterial hypertension led to its withdrawal from the market [52]. Only recently, a high-throughput drug screening study using *scn1Lab^−/−^* mutant zebrafish larvae, a validated model of DS, identified fenfluramine as promising candidates for repurposing in epilepsy [11,53]. In this preclinical study, fenfluramine significantly reduced zebrafish locomotor activity, as well as the frequency and duration of epileptiform discharges. Beyond seizure reduction, the anti-neuroinflammation and pro-survival effect of fenfluramine have been recently shown in a mammalian DS model [54]. Clinical efficacy has been demonstrated in multiple phase 3 randomized, placebo-controlled trials. In clinical studies, fenfluramine significantly reduced MCSF by 54% to 72.9% compared to placebo [55,56]. These findings were corroborated by open-label extension studies showing sustained seizure reduction and improvements in quality-of-life metrics. A more recent phase 3 trial further confirmed fenfluramine efficacy in DS, demonstrating a dose-dependent reduction in MCSF. Among participants receiving 0.7 mg/kg/day, 73% achieved a ≥50% reduction in seizures, and 48% achieved a ≥75% reduction, with significantly longer seizure-free intervals than placebo-treated patients [57]. Fenfluramine was well tolerated, with no reported cases of cardiac valvulopathy or pulmonary hypertension in any trial participant, as confirmed by regular echocardiographic monitoring. Real-world data from European cohorts, including Italy and Germany, have further supported fenfluramine safety and efficacy in DS clinical practice [10,12,58].

Although fenfluramine is approved only for patients aged ≥2 years, a recent prospective study evaluated its use in infants under 2 years with DS. In a median follow-up of 13 months, two of five patients achieved ≥50% seizure reduction, with one achieving seizure freedom for ≥6 months. Additionally, reductions in status epilepticus and seizure clusters were observed. No clinical or echocardiographic signs of valvular disease were detected, and none of the patients discontinued treatment due to adverse events [8]. Interestingly, long-term responders (≥50% seizure reduction) demonstrated improved executive function compared to non-responders. Neuropsychological assessments showed stable or improved developmental trajectories, with no evidence of regression and mild cognitive gains in 2 out of 5 patients. This encouraging outcome is especially relevant in an early onset syndrome typically associated with progressive developmental delay [8].

Like fenfluramine, lorcaserin, a selective 5-HT_2C_ receptor agonist, was originally approved by the FDA for weight management and was withdrawn from the market in 2020 following data suggesting a possible increased risk of cancer [59]. Despite this, interest in lorcaserin potential in treating epilepsy, particularly DEE, has continued. Like fenfluramine, lorcaserin was identified as a potential antiseizure agent in the *scn1Lab^−/−^* zebrafish DS model. At concentrations of 10–12.5 µM, lorcaserin significantly reduced both zebrafish locomotor activity and the frequency and duration of epileptiform discharges [60]. However, a phase 3 clinical trial to determine lorcaserin efficacy and safety as an adjunctive therapy for DS was recently discontinued [61]. Bexicaserin, another selective 5-HT_2C_ receptor agonist, showed sustained reductions in motor seizure frequency over two years and a favorable safety profile, in a phase 1/2 open label expanded access program involving DEE patients. The ongoing phase 3 trial will further assess bexicaserin’s efficacy and safety in a larger DEE cohort [4,7].

### 2.3. Cannabidiol

In 2018 cannabidiol (CBD) was approved by FDA and in 2019 by EMA for adjunctive treatment of seizures, in conjunction with clobazam, in DS and LGS in patients aged ≥2 years; since then, indications have expanded to include tuberous sclerosis complex. Cannabidiol is a non-psychoactive phytocannabinoid derived from *Cannabis sativa* exhibiting a broad spectrum of therapeutic effects [62]. CBD antiseizure potential is mediated by its ability to modulate neuronal excitability and neuroinflammation and grounds on a muti-target mechanism of action. This drug inhibits the GPR55 receptor which is implicated in excitatory neurotransmission and epileptogenesis; activates and subsequently desensitizes TRPV1 channels thus reducing neuronal hyperexcitability; inhibits the equilibrative nucleoside transporter 1 (ENT1) in microglia and astrocytes, thereby increasing extracellular adenosine and enhancing adenosine anticonvulsant effect; potentiates GABA_A_ receptor–mediated currents by interacting with an allosteric site [62]. Additional studies suggest that CBD modulates different ion channels including Nav1 neuronal sodium channels [63].

In preclinical models of DS, CBD significantly reduced seizure burden and improved autistic-like social deficits [13,64]. Following, CBD has undergone extensive evaluation in randomized controlled trials for DS. In a breakthrough phase 3, double-blind, placebo-controlled study involving 120 children and young adults with DS, adjunctive CBD (20 mg/kg/day) reduced MCSF from 12.4 to 5.9, compared with a smaller reduction in the placebo group (14.9 to 14.1). Although the ≥50% responder rate favored CBD (43% vs. 27%), this effect did not reach statistical significance in the primary analysis [65]. However, subsequent trials and open-label extensions confirmed significant and durable seizure reductions. A long-term extension study (median treatment duration of about 80 weeks) demonstrated a median 50% reduction in major motor seizures and 44% in total monthly seizures after 12 weeks, these effects being sustained for up to 96 weeks [66]. Among patients with DS or LGS, 53% showed ≥50% seizure reduction, 23% achieved ≥75%, and 6% became seizure-free for major motor seizures; respective rates for total seizures reduction were 46%, 26%, and 5%. Recent real-world studies have further supported the efficacy and safety of CBD in drug-resistant epilepsies, including DS, although responses vary and appear to be modulated by concomitant medications such as clobazam and valproate [67,68]. Common adverse events include somnolence (30%), diarrhea (24%), decreased appetite, and fatigue. Serious adverse events reported in long-term studies include convulsions (14%), status epilepticus (9%), pneumonia (5%), and fever (4%) [68]. Importantly, no new safety signals have emerged in post-marketing studies, and the rate of SUDEP remained consistent with background rates for severe epilepsies, with two cases reported in a large cohort over the observation period [68].

CBD is known to interact with multiple antiseizure drugs necessitating careful monitoring [69]. Due to inhibition of CYP2C19, co-administration of CBD with clobazam increases serum levels of the active metabolite nor-desmethylclobazam (norclobazam) principally metabolized by CYP2C19, which likely contributes to increased sedation. The combination of CBD and valproate has been associated with elevated liver transaminases and hyperammonemia, requiring routine monitoring of hepatic function and ammonia levels during co-therapy [70].

Despite other newer therapies like stiripentol or fenfluramine having demonstrated greater efficacy in terms of seizure reduction, CBD remains an important therapeutic option, particularly for drug-resistant patients [10].

### 2.4. Soticlestat

Soticlestat is a selective inhibitor of cholesterol 24-hydroxylase (CH24H or CYP46A1), an enzyme expressed predominantly in the brain that catalyzes the conversion of cholesterol to 24S-hydroxycholesterol (24S-HC). By reducing the amount of 24S-HC, soticlestat reduces glutamatergic excitatory transmission and attenuates neuroinflammation and glial activation, producing an anticonvulsant effect [71,72]. Inhibition of CH24H by soticlestat reduces central 24S-HC levels, a potent positive allosteric modulator of NMDA receptors, implicated in the pathogenesis of excitotoxicity and epileptogenesis [14,72]. Excess CH24H in astrocytes in epilepsy, aging, Alzheimer disease disrupts lipid rafts containing EEAT2, thus impairing glutamate uptake and increasing glutamate neurotoxicity. In addition, 24HC, via the integrin pathway, increases TNF-α and IL6. TNF-α enhances glutamate release and inhibits glutamate uptake, hence contributing to neural overexcitation. This novel mechanism of action was expected to provide seizure control with a distinct approach from current ASMs [17].

In preclinical studies, including *Scn1a*^+/−^ mouse models of DS, soticlestat significantly reduced seizure frequency, neural hyperexcitability, and SUDEP. Notably, it also conferred protection against hyperthermia-induced seizures, a hallmark of DS [14].

Early-phase clinical evaluation in patients with DEE initially provided evidence for efficacy. In a phase 1b/2a study, soticlestat adjunctive to standard of care resulted in 36.4% median reduction in seizure frequency, with an acceptable safety profile [73]. The subsequent phase 2 ELEKTRA trial, a randomized, double-blind, placebo-controlled study, assessed soticlestat as adjunctive therapy to standard of care in children with either DS or LGS. Among DS participants, soticlestat produced a statistically significant reduction in convulsive seizure frequency compared with placebo, whereas LGS patients experienced only a non-significant numerical decline in drop seizures, despite comparable reductions in 24S-HC levels across groups. In both cohorts, treatment was associated with improvements in global clinical functioning as assessed by caregiver and clinician impression scales. Common adverse events included lethargy and constipation, though tolerability was overall favorable [74,75]. The SKYLINE and SKYWAY multicenter, randomized, double-blind Phase 3 studies evaluated soticlestat plus standard of care versus placebo plus standard of care in patients with refractory DS and LGS, respectively. Soticlestat hardly missed the primary endpoint of reduction from baseline in convulsive seizure frequency and reduction from baseline in major motor drop seizure frequency as compared with placebo. Following these results in 2024, Takeda announced the discontinuation of the soticlestat development program in January 2025.

### 2.5. Zorevunersen (STK-001)

Zorevunersen (STK-001) is an ASO developed by Stoke Therapeutics using the TANGO (Targeted Augmentation of Nuclear Gene Output) platform. It has been designed to exclude an alternative poison exon (termed exon 20N), which introduces an in-frame premature stop codon into a subset of transcripts, resulting in non-productive mRNA and reduced Nav1.1 channel expression. Indeed, by sterically blocking exon 20N inclusion, zorevunersen reduces non-functional transcripts while enhancing productive *SCN1A* mRNA and restoring Nav1.1 protein levels from the wild-type allele [19,76]. In vitro, zorevunersen decreased the proportion of poison exon–containing transcripts from approximately 60% to under 5%. In wild-type neonatal mice (P2), intracerebroventricular (ICV) administration increased full-length *SCN1A* mRNA threefold along corresponding rise in Nav1.1 protein. Interestingly, in *Scn1a*-haploinsufficient mice, zorevunersen prevented the typical ~50% mortality by week 4, delayed seizure onset, decreased seizure frequency, reduced SUDEP, and improved interneuron excitability [19,20,21].

The Phase 1/2a open-label trials (MONARCH in USA, and ADMIRAL in UK) enrolled drug-resistant children and adolescents (ages 2–18) with genetically confirmed *SCN1A* variants, most receiving ≥3 antiseizure medications, with ~54% on ≥4 medications and half on fenfluramine. In dose cohorts receiving two or three 70 mg injections of zorevunersen, median convulsive seizure reduction was 85% at 3 months and 74% at 6 months post-last dose; lower reductions (~43–57%) were observed after a single 70 mg dose.

Open-label extension studies (SWALLOWTAIL and LONGWING) involving lower maintenance doses (30–45 mg every 4 months) demonstrated durable reductions in seizures over 12 months and improvements in cognition and behavior.

Approximately 30% of participants experienced treatment-emergent adverse events related to zorevunersen, primarily elevated cerebrospinal fluid protein and procedural vomiting; serious adverse events occurred in ~22%, though most were considered unrelated to the drug. In extension studies, cerebrospinal fluid protein elevations were more common (~74–79%), but did not exhibit clinical manifestations.

Zorevunersen has been granted breakthrough therapy designation by the FDA, as well as orphan drug designations from both the FDA and EMA. A global, randomized, double-blind, sham-controlled phase 3 trial (EMPEROR) was planned to commence mid-2025 to evaluate the efficacy, safety and tolerability of zorevunersen in children aged 2 to <18 with DS with a confirmed LOF variant in *SCN1A*. The trial is expected to enroll participants across the US, Japan, UK and EU. Participants will be randomized 1:1 to receive either zorevunersen via intrathecal administration or a sham comparator for a 52-week treatment period following an 8-week baseline period. Following the completion of the study, eligible participants will be offered ongoing treatment with zorevunersen as part of an open-label extension study. The primary endpoint of the study is percent change from baseline in major motor seizure frequency at week 28 in patients receiving zorevunersen as compared to sham. The key secondary endpoints are the durability of effect on major motor seizure frequency and improvements in behavior and cognition as measured by Vineland-3 subdomains, including expressive communication, receptive communication, interpersonal relationships, coping skills and personal skills. Additional endpoints include safety, Clinician Global Impression of Change, Caregiver Global Impression of Change, and the Bayley Scales of Infant Development [76].

As said before, the therapeutic strategy is applicable to DS patients with *SCN1A* LOF variants (e.g., truncating, nonsense, frameshift, or deletions) whose mutant alleles are subject to nonsense-mediated decay. It is contraindicated in patients with missense variants displaying GOF or dominant-negative effects, as increasing Nav1.1 expression may exacerbate disease pathology [77].

### 2.6. ETX101 (AAV9-RE^GABA^-eTF^SCN1A^)

ETX101 (AAV9-RE^GABA^-eTF*^SCN1A^*) is a gene regulatory therapy that delivers a transgene coding for an engineered *SCN1A*-specific transcription factor (eTF*^SCN1A^*) to upregulate the expression of the endogenous *SCN1A* gene. eTF binds to a conserved regulatory region upstream of the *SCN1A* transcription start site, enhancing *SCN1A* expression and protein translation. Expression of the transgene is controlled by a GABAergic inhibitory neuron-selective regulatory element (RE^GABA^). This approach increases the production of Na_V_1.1 at endogenous levels preferentially in GABAergic neurons, thereby restoring inhibitory function and minimizing potential off-target effects in excitatory cells [27]. ETX101 uses a clinically validated adeno-associated virus (AAV9) capsid and is delivered via ICV infusion in a one-time administration [17]. AAV-based vectors are considered safe as they cannot replicate or integrate into the genome. Local ICV administration of AAV-mediated gene therapy enhances delivery to the CNS, thereby increasing the potential for efficacy [5]. Preclinical data for ETX101 demonstrated, indeed, broad distribution into relevant CNS structures, including the cortex and hippocampus, in non-human primates (juvenile cynomolgus macaque), as well as long-term survival and reduction in seizure frequency and SUDEP in DS mouse models. No relevant adverse clinical or histopathological findings were reported [5,27].

POLARIS is the principal program designed to optimize and accelerate clinical development of ETX101 for the treatment of people with DS. This program includes phase 1/2 clinical studies to assess the safety and efficacy of ETX101 in infants and young children (ENDEAVOR, WAYFINDER, EXPEDITION). This investigational program builds on several initiatives, including Dravet ENGAGE study, which explored the needs and experiences of DS patients and families; ELUCIDATE, an ongoing biomarker discovery project aimed at identifying novel measures of disease progression; and ENVISION, the largest longitudinal natural history study of DS conducted to date, which provided insights into the disease course in infants and young children. ETX101 is currently in Phase 1/2 trials for infants and children with DS.

### 2.7. Additional ASO and Gene Editing Approaches

Recent advances in precision medicine for DS have identified a long antisense non-coding RNA, *SCN1A-dsAS* (downstream antisense), that negatively regulates *SCN1A* expression in human brain tissue, likely via transcriptional inhibition. Elevated levels of this antisense transcript correlate with reduced Nav1.1, contributing to the haploinsufficiency characteristic of DS [5]. To counter this, AntagoNAT ASOs targeting *SCN1A-dsAS* have been developed. In human DS patient fibroblasts, mouse models, and non-human primates, these ASOs upregulated *SCN1A* expression, restored Nav1.1 levels in fast-spiking inhibitory interneurons, normalized interneuron activity, and reduced seizure burden [7,23]. Further preclinical characterization of one of these ASOs, ASO3 (CO-3527), demonstrated efficacy in neuroblastoma-derived SK-N-AS cells [78]. ASO3 significantly downregulated *SCN1A-dsAS* transcripts and increased *SCN1A* mRNA levels, outdoing another ASO (Cur-1740-11), likely due to enhanced chemical stability and higher binding affinity [5]. These findings highlight the regulatory role of *SCN1A-dsAS* in *SCN1A* transcription and validate antisense-mediated modulation as a viable therapeutic strategy. By suppressing the inhibitory long non-coding RNAs, AntagoNATs can restore Nav1.1 expression and function, offering a novel precision approach for addressing the genetic alteration underlying DS.

Strategies based on genome editing have also been employed to upregulate *SCN1A* expression. The CRISPR/Cas9 system employs the Cas9 endonuclease, a molecular scissor, to introduce targeted double-strand breaks in DNA, enabling precise genome editing in animal, human, and plant cells [79]. A modified version of this system, in which the nuclease activity of Cas9 is inactivated (dCas9) and fused to transcriptional activator domains, can be directed to specific gene promoters to enhance gene expression. Targeting dCas9 fused to a transcriptional activator (*Scn1a*-dCas9A) to the *SCN1A* promoter has been shown to upregulate *SCN1A* transcription and Nav1.1 protein expression in primary mouse neurons isolated from *Scn1a*^+*/R1407X*^ DS pups [24]. In addition, DS mouse pups administered with the *Scn1a*-dCas9 system delivered ICV using a dual AAV9-based system showed increased firing rates in GABAergic interneuron and reduced hyperthermia-induced seizures, with an increased threshold for seizure induction. These results supported the therapeutic potential of this transcriptional activation strategy [24].

## 3. *SCN8A*-DEE

The *SCN8A* gene encodes the Nav1.6 voltage-gated sodium channel, a key determinant of neuronal excitability [1,80]. Nav1.6 is widely expressed throughout the brain, predominantly in excitatory neurons and, to a lesser extent, in inhibitory neurons, localizing primarily to the axon initial segment and nodes of Ranvier of myelinated axons. Like *SCN1A*, *SCN8A* expression is lowest during prenatal development and increases progressively toward adulthood [1]. Experimental studies in mice have demonstrated that selective deletion of Nav1.6 in cerebellar Purkinje neurons results in pronounced cognitive and motor deficits, highlighting the essential role of this channel in cerebellar and higher-order brain functions [81,82].

Clinically, *SCN8A*-related disorders exhibit a wide phenotypic spectrum, with disease manifestations classified into five groups based on age of onset and electroclinical biomarkers [83]. Epileptic phenotypes range from early-onset severe to moderate DEE, with severity influenced by the extent of developmental impairment and seizure refractoriness. Other phenotypes include Self-limiting familial infantile epilepsy (SeLFIE), also referred to as benign familial infantile epilepsy (BFIE), characterized by normal cognition and early-onset seizures that respond well to ASMs. Later-onset and no seizure phenotypes include Neurodevelopmental delay with generalized epilepsy (NDDwGE) and Neurodevelopmental delay without epilepsy (NDDwoE), which typically presents with mild to moderate intellectual disability (severe in ~10% of cases). Additional features may include hypotonia and movement disorders such as dystonia, ataxia, and choreoathetosis [7]. Importantly, *SCN8A* mutations are also associated with an increased risk of SUDEP. *SCN8A* variants can disrupt the normal function of Nav1.6 channels, leading to either GOF or LOF effects [1]. Most severe early-onset epilepsy have been associated with missense variants causing GOF and may respond to high-dose SCBs. GOF mutations often result in increased neuronal excitability due to altered channel kinetics, such as channels opening more readily, remaining open longer, or inactivating more slowly. Some mutations also generate persistent sodium currents between action potentials, further contributing to neuronal hyperexcitability. In contrast, LOF variants, presenting in individuals with intellectual disability without seizures, impair Nav1.6 function by reducing the number of channels or altering gating properties [1]. This leads to impaired action potential initiation and propagation and, consequently, disrupted synaptic communication, primarily during development. This early change might cause a persistent alteration in neuronal activity, leading to the autism spectrum disorder phenotype [81,82]. According to global consensus on the diagnosis and treatment of *SCN8A*-related disorders, SCBs, such as oxcarbazepine or carbamazepine, are the first-line treatment for GOF variants, severe DEE, mild/moderate DEE, and SeLFIE, whereas levetiracetam may worsen seizures and contribute to developmental regression in GOF patients. First-line treatment for NDDwGE is valproate, ethosuximide, or lamotrigine; SCBs are relatively contraindicated in LOF patients [83].

### 3.1. Relutrigine (PRAX 562)

Relutrigine is an orally active Nav1 blocker under development for the treatment of DEEs regardless of the etiology. This small molecule showed a preferential inhibition of persistent sodium current, a key driver of seizure symptoms in early-onset *SCN2A*-DEE and *SCN8A*-DEE and in other severe DEEs [15].

Relutrigine showed not only a 60-fold preference for persistent sodium current over peak sodium current but also a remarkable and unexpected 30-fold preference for use-dependent block over tonic block on Nav channels expressed in HEK-293 cell lines. Compared with available SCBs, such as lacosamide and carbamazepine, relutrigine showed an improved efficacy profile and improved tolerability, both in cell lines and animal models [4,84]. In vivo studies of relutrigine have demonstrated dose-dependent inhibition of seizures up to complete control in *SCN2A*, *SCN8A* and other DEE mouse models. The anticonvulsant activity of relutrigine was evaluated preclinically also in the MES (maximal electroshock seizure) animal model where it was again compared with standard SCBs carbamazepine and lamotrigine. Unlike carbamazepine and lamotrigine, the maximum effective dose of relutrigine (10 mg/kg) prevented seizures without affecting locomotor function. Regarding the safety profile, in preclinical MES models and spontaneous locomotor activity (sLMA) models, relutrigine showed a 3-fold higher protective index than carbamazepine and lamotrigine, although further studies are required to investigate the preclinical protective index of relutrigine after chronic treatment, which would reflect the planned clinical use.

Relutrigine has been generally well-tolerated in three phase 1 studies and has demonstrated biomarker changes indicative of Nav channel modulation. The EMBOLD study is a multicenter, randomized phase 2 clinical trial designed to explore the safety, tolerability, efficacy and pharmacokinetics of relutrigine in pediatric (aged 2–18 years) patients with early-onset *SCN2A*-DEE and *SCN8A*-DEE. EMBOLD cohort 1 study showed a placebo-adjusted monthly motor seizure reduction of 46% during the double-blind period and seizure freedom in over 30% of patients while on relutrigine. Additionally, significant improvements were observed in alertness, communication and seizure severity as noted by both clinicians and caregivers and 77% reduction in median seizure rate seen for patients in the long-term extension. At 11-month open-label extension period of the trial, an average of approximately 90% seizure reduction was observed in patients, and a sustained and continuous improvement in seizure-free periods was observed, with a mean of 67 days without seizures compared to 3 days in the baseline period. There were no new safety signals, drug related serious adverse events or dose reductions needed.

Based on the results of this study, the EMBOLD registrational cohort 2 is currently ongoing and continues to enroll, with initial results expected to come in the first half of 2026, followed by a potential new drug application filing [85].

Relutrigine has received orphan drug designation and rare pediatric disease designation from the FDA for the treatment of *SCN2A*-DEE, *SCN8A*-DEE and DS, as well as breakthrough therapy designation, and orphan drug designation from the EMA for the treatment of *SCN2A*-DEE and *SCN8A*-DEE. The breakthrough therapy designation from FDA was granted based on the highly compelling results from the phase 2 EMBOLD trial in *SCN2A*- and *SCN8A*-DEEs. Praxis has also recently initiated the EMERALD study, a registrational, 16-week, placebo-controlled trial evaluating relutrigine effect in seizure reduction in patients diagnosed with DEEs, regardless of etiology [86].

Thus, relutrigine demonstrated robust anticonvulsant activity in vivo, with good tolerability compared to other SCBs, suggesting potential for an improved clinical therapeutic window especially in patients where standard SCBs have limited efficacy due to poor tolerability. Given the role of persistent sodium current in modulating excitability, relutrigine has the potential to be a widely effective and well-tolerated ASM for both genetic and non-genetic epilepsies.

### 3.2. NBI-921352 (XEN901)

NBI-921352 is a selective inhibitor of Nav1.6 channels (IC50 ~ 0.051 µM) with high affinity towards the inactivated state of the channel and preferentially binding the voltage sensor domain of DIV. In a *SCN8A* GOF mouse model, NBI-921352 inhibited action potential generation in glutamatergic excitatory pyramidal neurons, bypassing GABAergic interneurons where Nav1.1 is dominant, and prevented electrically induced seizures [16]. Efficacy appeared superior to standard SCBs. Phase I clinical studies demonstrated that NBI-921352 was well tolerated at plasma concentrations above those required for efficacy in preclinical rodent studies, without signs of ataxia and motor symptoms. NBI-921352 is under development for both *SCN8A*-DEE epilepsy and adult focal seizures for oral administration. A phase II trial (KAYAK study) evaluating NBI-921352 in children and young adults with *SCN8A*-DEE is ongoing, with an estimated completion date in December 2025.

### 3.3. Additional ASO and RNA-Based Approaches

Additional strategies to treat *SCN8A*-DEE, still at the preclinical stage, include ASO and RNA-based therapies. Both repeated ASO therapy and a single AAV10-shRNA dose after seizure onset prevented recurrent seizures and extended survival over 12 months of follow-up in *SCN8A* mouse models. Indeed, in a conditional mouse model with Cre-dependent expression of the pathogenic *SCN8A* GOF mutation R1872W, an ASO targeting *Scn8a* downregulated *Scn8a* expression, extended lifespan and delayed seizure onset by 25 to 50% when administered after birth. This model exhibits early onset seizures, rapid progression, and 100% penetrance and survives for only 2 weeks [25]. Repeated ASO administration initiated after seizure onset, a timing more relevant to clinical treatment, provided sustained seizure control and significantly prolonged survival throughout the 12-month observation period. Similarly, a single neonatal treatment on postnatal day 1 with an adeno-associated virus serotype 10 (AAV10) vector expressing a short hairpin RNA (shRNA) targeting *Scn8a* (AAV10-shRNA) conferred long-term seizure protection and extended survival for up to one year [26]. These results support the disease-modifying potential of these approaches as a post-onset, long-term treatment for *SCN8A*-DEE.

### 3.4. Vormatrigine (PRAX 628)

Vormatrigine (PRAX-628) is a novel small molecule designed to reduce pathological neuronal hyperexcitability by targeting sodium channels in the brain, and it is currently under development as a once-daily, oral treatment for adult focal onset seizures and generalized epilepsy [18]. Vormatrigine exhibits a dual mechanism of action: selective modulation of persistent sodium current and enhanced use-dependent inhibition of peak sodium current. These properties enable precise targeting of hyperexcitable neuronal states while sparing normal activity. Pharmacological evaluations on different human Nav1 isoforms (Nav1.1, 1.2, 1.5, 1.6, 1.7, 1.8, and 1.9) revealed pan-Nav strong inhibitory activity. Even if vormatrigine has not been specifically developed for *SCN8A*-DEE, this drug induced significant suppression of Nav1.6 persistent sodium current (IC_50_ ~ 128 nM) and strong use-dependent block of peak sodium current, effects occurring at concentrations below those causing undesired tonic block. Vormatrigine was more potent compared with standard SCBs (e.g., carbamazepine, lamotrigine). These findings position this drug as a promising therapeutic candidate with a potentially superior safety and efficacy profile compared to existing SCBs [87,88]. Preclinical in vivo studies demonstrated the antiseizure efficacy of vormatrigine in the MES (maximal electroshock seizure) model, a translational model of focal epilepsy, at lower doses than standard-of-care ASMs. The in vivo anticonvulsant efficacy of vormatrigine (ED50 ~ 0.42 mg/kg) was approximately ten times higher than that of carbamazepine, cenobamate, lamotrigine and of the Kv7 opener XEN1101 (3.8–5.4 mg/kg). Data from the first cohort of patients in the RADIANT study demonstrated a robust seizure reduction and good safety profile [87,88]. The POWER1 study is enrolling.

## 4. *SCN2A*-DEE

The *SCN2A* gene encodes for the Nav1.2 voltage-gated sodium channel isoform widely expressed in developing and mature brain, especially in excitatory neurons [5]. As seen for other neuronal isoforms, variants in *SCN2A* have been associated with a broad spectrum of neurodevelopmental disorders, with or without epilepsy. Non-seizure phenotypes are characterized by severe encephalopathy, with developmental delays, movement disorders, schizophrenia, and various other conditions. *SCN2A* is also a candidate risk gene for autism spectrum disorder and nonsyndromic intellectual disability [1,28]. Seizure-related phenotypes include self-limited familial (inherited) neonatal–infantile epilepsy (SeLFNIE), previously called benign familial neonatal–infantile seizures (BFNIS), and early-infantile DEE such as Ohtahara syndrome, West syndrome, and LGS. *SCN2A* variants associated with neonatal and early infantile epilepsies generally produce increased Nav1 activity (GOF) and typically respond well to SCBs. Conversely, variants found in later-onset epilepsies and autism and intellectual disability without seizures cause LOF and show little to no response to SCBs. However, LOF *SCN2A* truncation can also occur in severe epilepsies including West syndrome and LGS. Thus, variant classification is not straightforward [28]. Different molecular consequences are associated with *SCN2A* LOF variants including reduced sodium current density, reduced protein expression, increased protein degradation, defects in trafficking, shifts in the voltage-dependent activation towards depolarized potentials, reduced probability of channel opening, and accelerated inactivation. GOF variants instead cause a shift in the voltage-dependent activation towards more hyperpolarized potentials, increased probability of channel opening, slowed inactivation, and persistent sodium current, leading to increased Nav1.2 channel activity [1].

In the absence of approved therapies for *SCN2A*-related disorders, clinical management is based on combination of ASMs and often drugs for managing debilitating comorbidities with several limitations [5,7]. However, a novel therapeutic approach based on ASO targeting *SCN2A* has been developed.

### Elsunersen (PRAX 222)

Elsunersen (PRAX-222) is an ASO designed to selectively reduce *SCN2A* gene expression, offering potential therapeutic benefit to patients with GOF *SCN2A* variants associated with early-onset *SCN2A*-DEE [5]. In vitro studies have demonstrated that elsunersen effectively decreases both *SCN2A* mRNA and protein levels. Preclinical in vivo studies using mouse models showed dose-dependent reductions in seizure frequency, improved behavioral and locomotor function, and increased survival rates. These data suggest the potential of elsunersen as the first disease-modifying treatment for *SCN2A*-DEE linked to GOF variants. The ongoing phase 1/2 EMBRAVE study represents the first-in-human clinical trial evaluating elsunersen safety and efficacy in pediatric patients (aged 2–18 years) with early-onset *SCN2A*-DEE [89]. Early clinical data of elsunersen in combination with SCBs in 5 patients indicated safety and seizure reduction, including cessation of previous refractory status epilepticus. In a single patient, the combination of intrathecal administration of elsunersen and relutrigine induced a significant reduction in seizures and status epilepticus highlighting the potential for complementary precision sodium channel modulation in early onset *SCN2A*-DEE [22,90]. Elsunersen has received orphan drug designation from both the FDA and EMA, as well as rare pediatric disease designation from the FDA for *SCN2A*-DEE treatment.

## 5. Challenges and Future Directions

The therapeutic management of DEEs is moving from a traditional, symptomatic therapy using combinations of conventional ASMs to disease-modifying approaches that either directly target the underlying etiology or exploits alternative pathways and aim for more thorough, sustained clinical improvement [4,28] (Figure 1; Table 1).

As multiple genetic and repurposed treatments become available, new clinical, methodological, and ethical challenges are expected to emerge [3].

The complex relationship between genotype and phenotype in many neurodevelopmental disorders requires precise variant interpretation to guide appropriate treatment and predict clinical outcomes. Both GOF and LOF variants within the same sodium channel gene have been identified, which can lead to markedly different phenotypes requiring different therapeutic strategies [29]. For instance, in *SCN*-related DEEs, GOF variants may necessitate gene-silencing strategies or specific SCBs, whereas LOF variants often result in haploinsufficiency phenotypes demanding increased protein expression [31]. Consequently, variant classification according to the functional defect is essential to inform therapeutic approaches aimed at upregulating, restoring, or suppressing gene or protein activity [91,92,93,94].

Understanding the natural history of the diseases and developing clinical biomarkers for deep phenotyping are fundamental to recognizing the populations most likely to benefit from specific therapies and to defining outcome measures for future clinical trials (e.g., ELUCIDATE and ENVISION studies for DS). Robust longitudinal and real-world evidence studies are essential to evaluate the long-term effectiveness and safety of both approved and investigational drugs in defined patient subgroups and to assess their impact on neurodevelopmental outcomes and other comorbidities [3]. In this regard, stiripentol and fenfluramine have shown the strongest seizure reductions in RCTs and network meta-analyses whereas cannabidiol is effective but, on average, less potent than the other two especially towards neurodevelopmental impairment [12,46,58]. Among novel small molecules, relutrigine and vormatrigine are next-generation Nav modulators designed to selectively inhibit persistent sodium current, a mechanism intended to improve both efficacy and tolerability compared with traditional SCBs. Relutrigine has shown promising clinical activity in *SCN2A*- and *SCN8A*-DEEs, with meaningful seizure reductions in some patients and better tolerability compared to older SCBs, despite RCT data remaining limited.

In addition, the definition of the optimal therapeutic window for intervention becomes relevant for maximizing clinical benefit. Many genetic epilepsies and neurodevelopmental disorders have early-onset and progressive courses, implying that treatment may be the most effective during defined developmental windows. Interventions initiated after these critical periods may have limited capacity to modify the overall trajectory of the disease. The attempt to administer fenfluramine off-label to children younger than 2 years highlights the need for timely intervention in DS [8,28].

Although ASO and gene therapy hold great promise for treating DEE and other neurodevelopmental disorders, several limitations must be overcome to fully exploit their potential [6,77]. Currently, ASO formulations may not readily cross the blood–brain barrier, requiring intrathecal or intracerebroventricular administration to achieve therapeutic CNS concentrations. While these delivery routes warrant targeted exposure, they are invasive and may carry procedural risks. Emerging strategies, such as nanoparticle encapsulation, receptor-mediated transcytosis, or chemical modification to enhance blood–brain barrier permeability, may enable less invasive and broader delivery. In addition, ASO pharmacokinetics pose challenges: their limited in vivo half-life necessitates repeated dosing, often multiple times per year, to maintain efficacy. The development of long-acting formulations could reduce treatment troubles and improve patient compliance. Similarly, AAV vectors face several key limitations. Their small packaging capacity (~4.7 kb) restricts use to smaller genes. Tissue-specific targeting and, conversely, off-target effects remain a technical challenge, limiting wide clinical translation [95]. In addition, immune and dose-related toxicities may raise safety concerns. To overcome these difficulties, emerging strategies include engineered capsids with improved trophism and immune evasion, novel promoters for tissue-specific delivery (e.g., AAV9 in ETX101) and cost-effective enhanced production platforms [95]. Despite these challenges, early-phase clinical data show promising seizures reduction and significant changes in cognition/behavioral impairment for some participants, consistent with disease-modifying intent of ASO and gene therapy approaches. These results have supported progression toward larger clinical trials.

In this framework, reliable cell and animal models of disease together with clinically oriented preclinical studies are essential to enhance the translatability of safety and efficacy data about new therapies to patients and de-risk clinical research [96,97]. In addition, improved knowledge of the mechanisms underlying *SCN*-related disorders, at the molecular, cellular and network levels in disease models, may allow for the identification of novel druggable pathways and/or facilitate drug repurposing [38,39]. For example, delayed GABA switch has emerged as a novel mechanism contributing to neurodevelopmental deficits in DS and a potential drug target [98].

Finally, the complexity involved in developing biological and innovative drugs presents significant challenges related to cost, time, long-term sustainability and equitable access, particularly for patients in low- and middle-income countries, that must be addressed in the future.

## Figures and Tables

**Table 1:** Recent therapeutic approaches for neuronal sodium channel disorders.

Therapeutic Strategy	Drug	Mechanism of Action	Drug Development Process	References
Small molecules	Stiripentol	GABA modulation and LDH inhibition	Approved for DS	[9,10]
	Fenfluramine	5-HT and sigma1 receptor modulation	Approved for DS, LGS	[11,12]
	Cannabidiol	GPR55 antagonism, TRPV1 agonism, adenosine transporter inhibition	Approved for DS, TSC	[13]
	Soticlestat	Cholesterol 24-hydroxylase inhibition	Discontinued	[14]
	Relutrigine (PRAX-562)	Use-dependent and persistent current Nav1.2/Nav1.6 block	Phase 3 EMERALD trial ongoing for DEE regardless of the cause	[15]
	NBI-921352 (XEN901)	Use-dependent selective Nav1.6 block	Phase 2 KAYAK trial ongoing for *SCN8A*-DEE	[16]
	Vormatrigine (PRAX-628)	Use-dependent and persistent current pan-Nav inhibition	Phase 3 RADIANT trial ongoing for adult focal onset epilepsy	[17,18]
ASO and RNA/DNA-based therapies	Zorevunersen (STK-001)	ASO, Exon 20N skipping targeting *SCN1A*	Phase 3 EMPEROR trial initiated for DS	[19,20,21]
	Elsunersen (PRAX-222)	Gapmer ASO; downregulation of *SCN2A*	Phase 1/2 EMBRAVE trial ongoing for GOF *SCN2A* DEE	[22]
	ASO targeting *SCN1A*-dsAS	Targeting long non-coding downstream RNA and increased expression of *SCN1A*	Preclinical stage for DS	[23]
	CRISPR/dCas9-based approach	Increased expression of *SCN1A*	Preclinical stage for DS	[24]
	ASO targeting *SCN8A*	Downregulation of *SCN8A*	Preclinical stage for GOF *SCN8A*-DEE	[25,26]
Gene therapy	ETX101 (AAV9-RE^GABA^-eTF*^SCN1A^*)	Regulatory gene therapy increasing expression of *SCN1A* in GABAergic interneurons	Phase 1/2 ENDEAVOR trial ongoing for DS	[17,27]

DS, Dravet syndrome; LGS, Lennox–Gastaut syndrome; TSC, Tuberous Sclerosis Complex; LDH, lactate dehydrogenase; ASO, antisense oligonucleotide.

## Data Availability

The original contributions presented in this study are included in the article. Further inquiries can be directed to the corresponding author.
